# Supplementary material for: The Clinical Utility of lncRNAs and Their Application as Molecular Biomarkers in Breast Cancer
Source: Int J Mol Sci. 2023 Apr 18;24(8):7426. doi: 10.3390/ijms24087426 (PMC10138835; doi:10.3390/ijms24087426)
Supplement: Supplementary file 1 [file ijms-24-07426-s001.zip › ijms-2272191-supplementary.pdf]

Supplementary Table S1. Molecular signature biomarkers based on lncRNAs for breast cancer.

LncRNAs	Disease	Number of samples	Application	Reference
MNX1-AS1, SIRLNT, AC092920.1, AC105219.1, AL355312.3, AC055854.1, LINC01117 and *ACTA2-AS1*	Breast Cancer	Discovery phase: 808 tumor tissues	Tumor Status (p-value < 0.001)<br>Stage (p-value < 0.001)<br>Node status (p-value < 0.001)	[172]
RP5-1198O20	Breast cancer	Discovery phase: 915 tumor tissues and 85 normal tissues.<br>Replication phase: 50 tumor tissues and 23 normal tissues.	Overall survival (p-value=0.041)	[14]
CAT104, LINC01234 and *STXBP5-AS1*	Breast cancer	Discovery phase: 1064 tumor tissues (532 for training set and 532 for validation set).<br>Replication phase: 104 tumor tissues and 17 normal tissues.	Overall survival (p-value<0.001)	[61]
AC010343.3, AL354793.1 and *FGF10-AS1*	Triple negative breast cancer	Discovery phase: 1181 samples, 308 triple negative breast cancer, 760 non triple negative breast cancer and 113 normal tissues (590 for training set and 591 for validation set).	Pathologic stage (p-value=2.9e-06)<br>5-year survival (p-value<0.001)	[173]
P11-482H16.1, AC010729.1, RP11-983P16.4, FOXD3-AS1, LINC01249, AC096574.4, AC015971.2, AC012487.2 and *RP11-15A1.2*	Breast cancer	Discovery phase: 508 tumor tissues (254 for training dataset and 254 for validation set).<br>Replication phase: 916 tumor tissues.	Metastasis-free survival (p-value<0.03)	[174]
FOXD2-AS1, A1BG-AS1, C9orf163, GSN-AS1 and *LINC00893*	Breast cancer	Discovery phase: 318 tumor tissues and 292 normal tissues.<br>Replication phase: 241 tumor tissues	Relapse-free survival (p-value=0.001)<br>Pathological stage (p-value=0.003)<br>Tumor status (p-value<0.02)	[175]
A1BG-AS1, AC004477.3, AC004585.1 and *AC004854.2*	Breast cancer	Discovery phase: 455 tumor tissues.<br>Validation phase: 456 tumor tissues.	Relapse-free survival (p-value<0.0001)	[176]
PINK1-AS, RP11-259N19.1, KLF3-AS1, LINC00339, LINC00472, RP11-351I21.11, KB-1460A1.5 PKD1P6-NPIPP1, PDCD4-AS1, KLF3-AS1, PP14571 and *RP11-69E11.4*	Breast cancer ER positive	Discovery phase: 298 tumor tissues.<br>Validation phase: 160 tumor tissues.	Relapse-free survival (p-value=0.005)	[177]
LINC00963, LOC100507388, LINC00094, SMG7-AS1, PP14571, ASAP1-IT1, LOC103344931, FAM182A and *HCG26*	Breast cancer ER positive	Discovery phase: 310 tumor tissues.<br>Validation phase: 198 tumor tissues.	Pathological complete response (p-value=0.002)<br>Chemotherapy response (p-value=0.046)<br>5-years distant relapse-free survival (p-	[178]

			value<0.05)	
AC007686.3, BAIAP2-DT, LINC00926, LINC01016 and *MAPT-AS1*	Breast cancer	Discovery phase: 1903 tumor tissues. Validation phase: 327 tumor tissues.	5-year survival (p-value<0.05)	[179]
RP11-524D16_A.3, HOTAIR, AL645608.1, TSPOAP1-AS1 and *RP11-13L2.4*	Breast cancer	Discovery phase: 327 tumor tissues. Validation phase: 564 tumor tissues.	5-year disease-free survival (p-value=3.29e-05)	[180]
AC106782.5, AC106782.5, AP000695.1, SIAH2-AS1, AC084125.4, POLH-AS1, AC005041.3, AP001528.1, SNHG25, AC110995.1, LINC01094, LINC01235, LINC01819, AC009171.2, LINC00922, MEG3, MIR22HG, AC109361.2, LINC01703, AC004687.1, LINC02613, AC003965.2, AL139246.5, AC004585.1, TDRKH-AS1, MIR99AHG, LINC02544, AP001453.4, LINC01615, AC092119.2, LINC01569, AL121832.2, HSD11B1-AS1, RHPN1-AS1, AC124319.1, AC022146.2, AL356417.2, LINC00511, U62317.4, AC103760.1, NR4A1AS, YTHDF3-AS1, MAGI2-AS3, TFAP2A-AS1, AL121790.2, AC040169.1, AC147067.2, AP005131.7, AP005131.2, U62317.1, AGAP1-IT1, ARHGAP29-AS1, AC040970.1, AC144450.1, AL645608.7, USP30-AS1, U73166.1, AL589765.4, LMNTD2-AS1, AC138904.1, AP005131.3, AC022007.1 and *AC109322.1*	Breast cancer	Discovery phase: 1103 tumor tissues and 113 normal tissues.	Overall survival (p-value<0.001)	[181]
RP11-890B15.3, RP11-1024P17.1, MFI2-AS1 and *RP11-180N14.1*	Triple negative breast cancer	Discovery phase: 98 tumor tissues. Validation phase: 107 tumor tissues.	Overall survival (p-value<0.02)	[182]
AC010834.3, AL031186.1, AL136531.1, LINC01871, MAPT-AS1, SEMA3B-AS1 and *AL606834.2*	Breast cancer	Discovery phase: 848 tumor tissues.	5-year survival (p-value<0.05)	[183]
TFAP2A-AS1, CHRM3-AS2, MIAT, DIAPH2-AS1, NIFK-AS1, LINC00472, MEF2C-AS1 and *WEE2-AS1*	Breast cancer	Discovery phase: 327 tumor tissues (for training set) and 1194 tumor tissues (for validation set).	Overall survival (p-value<0.001)	[184]
U79277, AK024118, BC040204 and *AK000974*	Breast cancer	Discovery phase: 327 tumor tissues (for training set) and 560 tumor tissues (for validation set).	Disease-free survival (p-value<0.01)	[185]
LINC00472, SNHG17, RPARP-AS1, lnc-INSIG2-6, lnc-ECD-2 and *lnc-HLA-DRB1-5*	Breast cancer	Discovery phase: 4813 tumor tissues.	Disease-free survival (p-value<0.001)	[186]
OTUD6B-AS1, AL122010.1, AC136475.2, AL161646.1, AC245297.3, LINC00578,	Breast cancer	Discovery phase: 1053 tumor tissues and 111 normal tissues.	Overall survival (p-value<0.001)	[187]

LINC01871 and *AP000442.2*				
MAPT-AS1, LINC01871, AL122010.1, AC090912.1 and *AC061992.1*	Breast cancer	Discovery phase: 1108 tumor tissues.	Overall survival (p-value<0.001)	[188]
CYTOR, LMNTD2-AS1, LYPLAL1-AS1, USP30-AS1, RHPN1, LINC01655, AP005131.2, AC004988.1 and *AC079289.3*	Breast cancer	Discovery phase: 1096 tumor tissues and 112 normal tissues.	Overall survival (p-value<0.001)	[189]
MAPT-IT1, SLC26A4-AS1, VPS9D1-AS1, PCAT18, LINC01234, SPATA41 and *LINC01215*	Breast cancer	Discovery phase: 486 tumor tissues (for training set) and 487 tumor tissues (for validation set). Validation phase: 3409 tumor tissues.	Overall survival (p-value<0.001)	[190]
Z68871.1, LINC00578, AC097639.1, AP003119.3, AP001207.3, LINC00668, AL122010.1, AC245297.3, LINC01871, AP000851.2, AC022509.2 and *SEMA3B-AS1*	Breast cancer	Discovery phase: 1053 tumor tissues.	Overall survival (p-value=3.021e-11)	[191]
SH3BP5-AS1, AC012073.1, AC120114.1, LINC00377, AL133467.1, AC036108.3 and *AC020663.2*	Breast cancer	Discovery phase: 1086 tumor tissues and 79 normAbbreviationsal tissues.	Overall survival (p-value<0.001)	[192]

Supplementary Table S2. Clinical trials for lncRNAs in cancer.

Clinical Trial ID	Title	Disease	Number of participants	Phase
NCT05334849	Prospectively Predict the Immunotherapy Response of Gastric Cancer Based on Circulating Exosomal LncRNA-GC1 Biopsy	Advanced Gastric Carcinoma	80	Not Applicable
NCT05397548	Use of Circulating Exosomal LncRNA-GC1 to Monitor Gastric Cancer (UCELMGC)	Gastric Cancer	700	Not Applicable
NCT05647941	Neo-adjuvant Chemotherapy Evluation in Gastric Cancer Patients Based on Circulating Exosomal LncRNA-GC1	Gastric Cancer	700	Not Applicable
NCT03738319	Non-coding RNA in the Exosome of the Epithelia Ovarian Cancer	High Grade Serous Carcinoma Ovarian Cancer	160	Not Applicable
NCT05270174	A Prospective, Multicenter Cohort Study of Urinary Exosome lncRNAs for Preoperative Diagnosis of Lymphatic Metastasis in Patients With Bladder Cancer	Bladder Cancer	74	Not Applicable
NCT02641847	TA(E)C-GP Versus A(E)C-T for the High Risk TNBC Patients and Validation of the mRNA-lncRNA Signature.	Triple Negative Breast Cancer, Breast Cancer.	503	Phase 2 Phase 3
NCT04767750	Role of LncRNA H19 in The Regulation of IGF-1R Expression	Hepatocellular Carcinoma Type 2 Diabetes Cancer	101	Not Applicable
NCT03057171	A Study on the Gastrointestinal Disease and Helicobacter Pylori Controlled Long Non-coding RNA	Helicobacter Pylori Infection Gastric Ulcer Duodenal Ulcer Stomach Cancer	50	Not Applicable
NCT03830619	Serum Exosomal Long Noncoding RNAs as Potential Biomarkers for Lung Cancer Diagnosis	Lung Cancer	1000	Not Applicable
NCT03742856	A Multi-omics Study of Epithelial Ovarian Cancer	Epithelial Ovarian Cancer	80	Not Applicable
NCT04946968	Phase-2 Dacomitinib Study on Patients With EGFR-Driven Advanced Solid Tumours With Low EGFR-AS1 IncRNA Expr or Other Novel Emerging Biomarkers	Advanced Solid Tumours Non-small Cell Lung Cancer Head and Neck Squamous Cell Carcinoma	104	Phase 2
NCT03742869	HPV Integration and Tumorigenesis of Uterine Cervical Adenocarcinoma	Cervical Adenocarcinoma Human Papillomavirus	40	Not Applicable

NCT04010487	Multi-omics Study on the Pathogenesis of Malignant Transformation of Adenomyosis	Adenomyosis Endometrial Cancer	40	Not Applicable
NCT02221999	Weekly Paclitaxel and Cisplatin to Treat Hormone Receptor Positive and Triple Negative Breast Cancer Patients (SHPD002).	Tubular Breast Cancer, Mucinous Breast Cancer, Invasive Ductal Breast Cancer, Inflammatory Breast Cancer.	250	Phase 2 Phase 3
NCT04269746	Assessment Of Long Noncoding RNA CCAT1 In Colorectal Cancer Patients	Colorectal Cancer	100	Not Applicable
NCT03469544	Long Non Coding RNA HOTAIR and Midkine as Biomarkers in Thyroid Cancer	Thyroid Cancer	90	Not Applicable
NCT02948855	Regulation of LncRNA For Breg in Patients With Thymoma and Autoimmune Diseases	Thymoma Autoimmune Disease	0	Not Applicable
NCT04729855	Association of Autophagy-related Genes ,LncRNA and SNPs With Colorectal Cancer in Egyptian Population	Colo-rectal Cancer	148	Not Applicable
NCT05577364	Selinexor in Combination With R-CHOP Followed by Selinexor Maintenance for Untreated EBV-positive DLBCL Patients	EBV-Positive Diffuse Large B-Cell Lymphoma, Nos	54	Phase 1 Phase 2
NCT04946266	Prospective Validation of the Prognostic Value of Long Non-coding MFI2-AS1 RNA in Localized Clear Cell Kidney Cancers (MFI2-PREDICT)	Kidney Cancer	260	Not Applicable
NCT05141383	Comparative Study of Diagnostic and Prognosis Biomarkers of Prostate Cancer in Liquid Biopsy (HOPE)	Prostate Cancer	118	Not Applicable
NCT01632930	Medical Economics of Urinary PCA3 Test for Prostate Cancer Diagnosis	Patients Scheduled for Prostate Biopsy Because of Increased Serum PSA and/or Abnormal Digital Rectal Examination	962	Not Applicable
NCT01024959	Clinical Evaluation of the PROGENSA(Registered Trademark) Prostate Cancer Gene 3 (PCA3) Assay in Men With a Previous Negative Biopsy Result (PCA3)	Prostatic Neoplasms	507	Not Applicable
NCT01177436	Prostate Cancer Antigen 3 (PCA-3) Gene Project	Prostate Cancer Benign Prostatic Hypertrophy	31	Not Applicable
NCT01020448	Effect of Triptorelin (Decapeptyl®) 22.5 mg on Two Biomarkers in Patients With Advanced Prostate Cancer (Triptocare)	Prostate Cancer	339	Phase 3
NCT05088811	The Role of Long Non-coding RNAs WRAP53 and UCA-1 as	Hepatocellular Carcinoma	80	Not Applicable

	Potential Biomarkers in Diagnosis of Hepatocellular Carcinoma	Liver Cirrhosis		
NCT00711997	Phase 1/2a DTA-H19 in Patients With Unresectable Pancreatic Cancer	Pancreatic Neoplasms	9	Phase 1<br>Phase 2
NCT04767750	Role of LncRNA H19 in The Regulation of IGF-1R Expression	Hepatocellular Carcinoma<br>Type 2 Diabetes<br>Cancer	101	Not Applicable
NCT00393809	Safety and Proof of Concept Study of Intravesical DTA-H19 in Patients With Superficial Bladder Cancer	Bladder Neoplasms	19	Phase 1<br>Phase 2
NCT00826150	Phase 1/2a Study of DTA-H19 in Advanced Stage Ovarian Cancer	Ovarian Cancer	14	Phase 1<br>Phase 2
NCT00595088	Phase 2b, Trial of Intravesical DTA-H19/PEI in Patients With Intermediate-Risk Superficial Bladder Cancer	Superficial Bladder Cancer	47	Phase 2

[*] Clinical trials were consulted in https://clinicaltrials.gov/ (last accessed January 13th 2023).
Search terms: [LncRNA] AND [Cancer].
